# Fabrication and Optimization of Bilayered Nanoporous Anodic Alumina Structures as Multi-Point Interferometric Sensing Platform

**DOI:** 10.3390/s18020470

**Published:** 2018-02-06

**Authors:** Mahdieh Nemati, Abel Santos, Dusan Losic

**Affiliations:** 1 School of Chemical Engineering, The University of Adelaide, Engineering North Building, Adelaide 5005, Australia; mahdieh.nemati@adelaide.edu.au (M.N.); abel.santos@adelaide.edu.au (A.S.); 2 Institute for Photonics and Advanced Sensing (IPAS), The University of Adelaide, Adelaide 5005, Australia; 3 ARC Centre of Excellence for Nanoscale BioPhotonics (CNBP), The University of Adelaide, Adelaide 5005, Australia

**Keywords:** nanoporous anodic alumina, structural fabrication, reflectometric interference spectroscopy, optical sensors, biomolecule detection

## Abstract

Herein, we present an innovative strategy for optimizing hierarchical structures of nanoporous anodic alumina (NAA) to advance their optical sensing performance toward multi-analyte biosensing. This approach is based on the fabrication of multilayered NAA and the formation of differential effective medium of their structure by controlling three fabrication parameters (i.e., anodization steps, anodization time, and pore widening time). The rationale of the proposed concept is that interferometric bilayered NAA (BL-NAA), which features two layers of different pore diameters, can provide distinct reflectometric interference spectroscopy (RIfS) signatures for each layer within the NAA structure and can therefore potentially be used for multi-point biosensing. This paper presents the structural fabrication of layered NAA structures, and the optimization and evaluation of their RIfS optical sensing performance through changes in the effective optical thickness (EOT) using quercetin as a model molecule. The bilayered or funnel-like NAA structures were designed with the aim of characterizing the sensitivity of both layers of quercetin molecules using RIfS and exploring the potential of these photonic structures, featuring different pore diameters, for simultaneous size-exclusion and multi-analyte optical biosensing. The sensing performance of the prepared NAA platforms was examined by real-time screening of binding reactions between human serum albumin (HSA)-modified NAA (i.e., sensing element) and quercetin (i.e., analyte). BL-NAAs display a complex optical interference spectrum, which can be resolved by fast Fourier transform (FFT) to monitor the EOT changes, where three distinctive peaks were revealed corresponding to the top, bottom, and total layer within the BL-NAA structures. The spectral shifts of these three characteristic peaks were used as sensing signals to monitor the binding events in each NAA pore in real-time upon exposure to different concentrations of quercetin. The multi-point sensing performance of BL-NAAs was determined for each pore layer, with an average sensitivity and low limit of detection of 600 nm (mg mL^−1^)^−1^ and 0.14 mg mL^−1^, respectively. BL-NAAs photonic structures have the capability to be used as platforms for multi-point RIfS sensing of biomolecules that can be further extended for simultaneous size-exclusion separation and multi-analyte sensing using these bilayered nanostructures.

## 1. Introduction

There are an increasing number of research studies focused on exploring new nanoporous materials and their applications in highly sensitive chemical and biosensing devices which are cost-effective and more sensitive due to their specific surface areas and the unique properties of their pore structures. Among them, nanoporous anodic alumina (NAA) fabricated by electrochemical anodization has been extensively explored for optical transduction systems in a broad range of sensing applications including biomedical, pharmaceutical, industrial, and environmental [1]. The versatile nanoporous structure of NAA makes it an excellent optical platform to develop sensing devices based on broad ranges of detection principles including UV-Vis, surface plasmon resonance, reflective interference, optical waveguiding, Raman spectroscopy and others [2,3,4]. NAA pore structures are highly sensitive to any alteration of the effective medium including the binding of molecules, which can be subsequently translated into readable optical signals [5,6,7,8]. Reflectometric interference spectroscopy (RIfS) is one of most attractive optical sensing techniques used for development of low cost and simple sensing devices [9,10,11,12]. The method is based on interaction of optical platforms and light reflection where reflected light is amplified at specific wavelengths with enhanced reflections, creating characteristic interferometric spectra due to the Fabry-Pérot effect. In NAAs, the Fabry-Pérot effect can be described by Equation (1),

EOT = 2n_eff_ Lcos θ = *m*λ, (1)

where EOT is the effective optical thickness of the NAA film, n_eff_ is its effective refractive index, L is its physical thickness, *m* is the order of wavelength oscillation in the spectrum of RIfS, λ is maximum wavelength, and θ is the incidence angle of light. This expression can be used to quantify changes in the effective medium of the NAA platform, using effective optical thickness changes (∆EOT) as the sensing parameter [13]. RIfS using optical platforms were extensively explored by the Gauglitz group and others to detect a broad range of analytes including organic molecules, gases, DNA, pesticides, etc. [14,15,16,17]. 

In contrast to RIfS, systems based on solid optical platforms, the combination of RIfS with nanoporous platforms such as NAA has a set of attractive properties such as a high effective surface area which increases the number of binding centres and effective medium that can be fabricated with precision to create NAA structures with highly sensitive sensing platforms [1,18]. Our previous studies demonstrated that the NAA can be successfully used for the development of highly sensitive RIfS devices for a broad range of applications including organic molecules, enzymes, drugs, cancer cells, etc. [13,19,20,21,22]. To advance sensing and optical (RIfS) performance of these NAA platforms many strategies have been employed using structural fabrication methods to optimise pore structures, introduce appropriate surface modifications and design of RIfS sensing devices combining with microfluidics [23,24]. Several studies have demonstrated that the nanoporous structure of NAA can be precisely fabricated by different anodization approaches to generate multi-dimensional photonic crystal structures such as distributed Bragg reflectors and funnel-like photonic films that can significantly improve their sensing performance [25,26]. Marshal’s group and others recently demonstrated that funnel-like NAA structures (multi-layered NAA films featuring a decreasing pore diameter from top to bottom) can provide characteristic RIfS properties that could offer new sensing applications [27,28,29,30]. It is indicated that bilayered NAA (BL-NAA) nanostructures feature a complex RIfS spectrum, which can be resolved by Fourier transform (FFT) to discern characteristic peaks associated with each layer and be independently utilized to monitor and quantify molecular binding events occurring in each layer within the BL-NAA structure [31,32,33]. However, more fundamental and applied studies are needed in order to fully understand RIfS characteristics of bi-layered and multi-layered NAA structures and exploit their potential for multi-point and multi sensing applications. 

In this study, we fabricated bi-layered NAA films (BL-NAA) with hierarchical funnel-like structures in order to explore their RIfS performances and potential to be used for advance biosensing, including multi-analyte detection. Our fabrication approach consisted of several sequential anodization and chemical etching steps, which enable the design of bi-layered optical structures with a specific pore diameter and length in depth. It is proposed that fabricated BL-NAA structures will feature three distinct and well-resolved peaks in their FFT spectrum that correspond to each physical layer (top layer I, bottom layer II, and total layer III). These characteristic peaks are proposed for independent molecular sensing inside pores in order to explore their simultaneous size-exclusion and multi analytes detection. Quercetin is a plant flavonoid present in various fruits, vegetables and medicinal herbs [34] that has as antioxidant [35,36] properties with the potential for therapeutic applications such as neuroprotective effect, cardiovascular protection, anti-cancer, and anti-inflammatory properties [37,38,39]. To achieve selectivity, the surface chemistry was functionalized with human serum albumin (HSA) attached on 3-aminopropyl) trimethoxysilane (APTES) in order to endow these with chemical selectivity towards polyphenol quercetin. Binding events between quercetin and HSA molecules are measured in real-time measurements through changes of effective optical thickness (EOT) of each layer in the FFT spectrum of BL-NAA by reflective interferometric spectroscopy (RIfS), enabling the assessment of the optical sensing performance of BL-NAAs platform. A systematic study analysing the effect of the geometric features of BL-NAA on the sensitivity and low limit of detection was performed in order to determine the limitations and potential of this system for multi-analyte and size-exclusion biosensing. 

## 2. Materials and Methods

### 2.1. Materials 

High purity (99.9997%) aluminum foils (Al) 0.32 mm thick were supplied by Goodfellow Cambridge Ltd. (Huntingdon, UK). Oxalic acid (C_2_H_2_O_4_), perchloric acid (HClO_4_), phosphoric acid (H_3_PO_4_), chromic acid (H_2_CrO_4_), (3-aminopropyl)trimethoxysilane (APTES), hydrogen peroxide (H_2_O_2_), glutaraldehyde (CH_2_(CH_2_CHO)_2_—GTA), sodium hydroxide (NaOH), human serum albumin (HSA), quercetin (C_15_H_10_O_7_), and phosphate buffer saline (PBS) were purchased from Sigma-Aldrich (Australia). Ultrapure water from Option Q–Purelabs (Australia) was used for preparing the aqueous solutions used in this study.

### 2.2. Fabrication of Bilayered NAA Films (BL-NAAs) and NAAs

**Figure 1:**
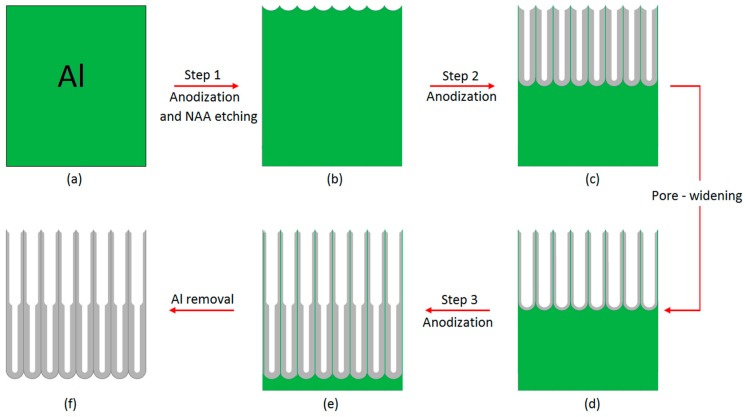
Fabrication process of interferometric bilayered nanoporous anodic alumina (BL-NAA) structures. (**a**) Electro polished high purity aluminum; (**b**) First step of anodization and resulting chemically etched nanostructure; (**c**) Second step of anodization results top layer of BL-NAAs; (**d**) Pore-widening leads to nanopores with a larger pore diameter; (**e**) Third step of anodization results in the bottom layer of the nanopore structure which has a smaller pore diameter compared to top layer; (**f**) Supporting aluminum was removed from the backside of platform. * Note that, stage (**e**) was not included for nanoporous anodic alumina (NAAs) fabrication.

Al samples were anodized in an electrochemical cell by a multiple step anodization process. Al samples were first sonicated in ethanol to remove organic residue and electrochemically polished in a mixture of EtOH: HClO_4_ 4:1 (v:v) at 20 V and 5 °C for 3 min to achieve a smooth surface. The first anodization step was performed in a solution of 0.3 M H_2_C_2_O_4_ at 40 V and 5 °C for 20 h. After that, the NAA structure was etched in an aqueous solution of 0.2 M H_2_CrO_4_ and 0.4 M H_3_PO_4_ at 70 °C for 3 h. Two anodization steps were carried out using 0.3 M H_2_C_2_O_4_ at 40 V and 5 °C for different duration. The fabrication protocol for each BL-NAA platform is detailed in Table 1. A pore widening treatment between anodization steps was used to increase the diameter of the nanopores. This process was carried out by wet chemical etching in an aqueous solution of H_3_PO_4_ 5 wt. % at 35 °C for 15 min (Table 1). Figure 1 shows a generic illustration of the fabrication process used to produce BL-NAA. We fabricated two control NAAs wer under the same anodization conditions featuring straight nanopores from top to bottom at 0 and 15 in of pore widening treatment (Table 1).

### 2.3. Surface Chemistry Functionalization of BL-NAAs and NAAs

The fabricated BL-NAAs and NAAs were chemically functionalized with APTES following a well-established protocol [1,40]. In brief, hydroxyl groups were created on the inner surface of BL-NAAs and NAAs by immersion in 30 wt. % H_2_O_2_ at 90 °C over 15 min. After that, silane molecules were immobilized onto the inner surface of BL-NAA nanopores by chemical vapor deposition method. This process was carried out under vacuum condition at 135 °C for 3 h. Finally, BL-NAAs and NAAs were coated with an ultrathin gold film (i.e., 5 nm) deposited by a sputter coater (sputter coater 108auto, Cressington, Redding, CA, USA) in order to enhance the light interference as reported elsewhere [41]. 

### 2.4. Optical Sensitivity Assessment of BL-NAAs and NAAs by RIfS

The sensing performance of BL-NAAs and NAAs was assessed by measuring changes of the EOT in each optical layer using quercetin as a sensing agent model to establish the sensing parameters including sensitivity (*S*), linearity (*R*^2^), and low limit of detection (*LLOD*). These sensing parameters were estimated by measuring shifts in the EOT of each layer according to Equation (1).

Real-time screening of EOT shifts was performed using a RIfS system combined with a cell flow system. The APTES functionalized BL-NAAs and NAAs were placed into the flow cell, where phosphate buffer saline (PBS) was allowed to flow until a stable baseline was achieved. In order to immobilize HSA onto the surface, 2.5% glutaraldehyde was used as a cross-linker and flowed through the system for 30 min over BL-NAAs and NAAs. GTA molecules activate the amine functional group (-NH_2_) of APTES molecules inside nanopores of BL-NAAs and NAAs. 

PBS was flowed again for 15 min to wash non-covalent bound of GTA molecules. After this, 1 mg mL^−1^ human serum albumin (HSA) solution was allowed to flow through the system for 1.5 h in order to immobilize HSA onto the inner surface of BL-NAA and NAAs. Next, PBS solution was allowed to flow for 15 min to remove physiosorbed HSA molecules. After that, analytical solutions of the analyte agent, quercetin, with different concentrations (i.e., 0.05, 0.125, 0.25, 0.375, 0.5, 1 mM) were allowed to flow through the system untill a stable line was achieved confirming that all HSA molecules were saturated with quercetin. Finally, PBS solution was let flow to establish the total EOT changes associated to HSA-Quercetin binding. Note that, this process was carried out at pH 7.5 and room temperature.

### 2.5. Structural Characterization of BL-NAAF and NAAs

The structural characteristics of the prepared BL-NAAs and NAAs (top and fractured structures) were established by field emission scanning electron microscopy (FEG-SEM FEI Quanta 450, ThermoFisher Scientific, OR, USA). Imaging was performed at least on four different spots for each sample in order to probe the reproducibility of process and uniformity of the fabricated structures. 

## 3. Results and Discussion

### 3.1. Structural Characterisation of Prepared BL-NAAs and NAAs

**Figure 2:**
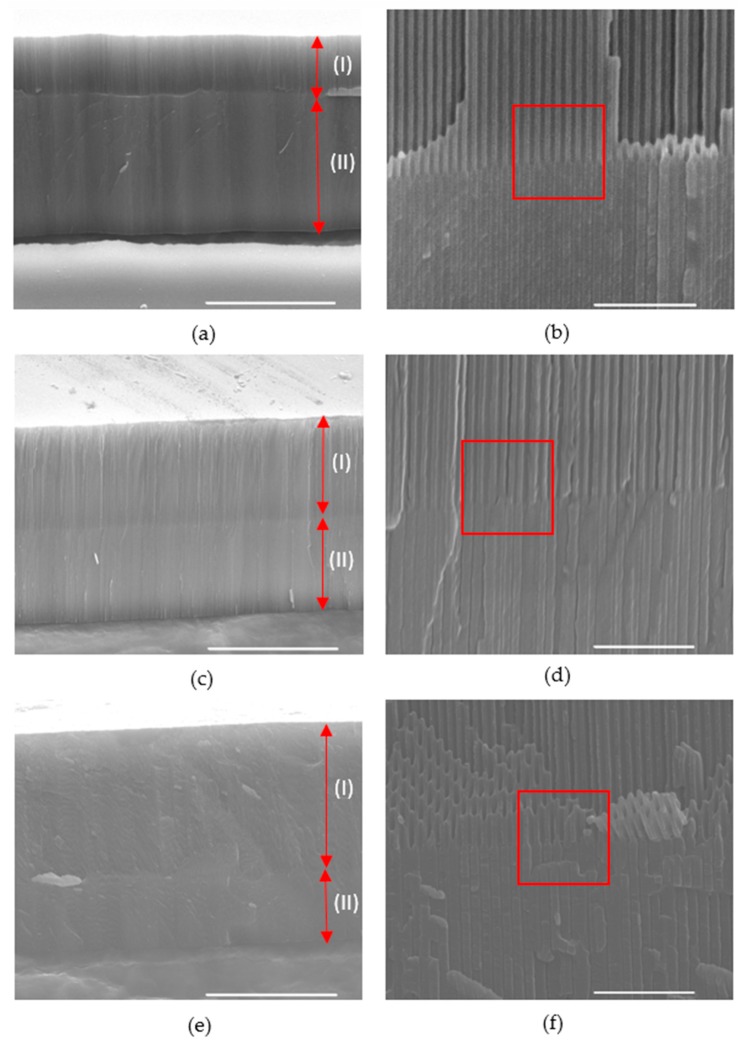
Scanning electron microscopy (SEM) structural characterization of fabricated BL-NAAs showing the length of pore layers and pore diameters. (**a**) Cross-section view of BL-NAA_(25/75)_, scale bar is 20 μm. (**b**) Cross-section view of BL-NAA_(25/75)_, scale bar is 1 μm. (**c**) Cross-section view of BL-NAA_(50/50)_, scale bar is 20 μm. (**d**) Cross-section view of BL-NAA_(50/50)_, scale bar is 1 μm. (**e**) Cross-section view of BL-NAA_(75/25)_, scale bar is 20 μm. (**f**) Cross-section view of BL-NAA_(75/25)_, scale bar is 1 μm.

The morphology of BL-NAAs prepared using different anodization conditions to make materials with different thicknesses and pore diameters of each layer and control NAAs structures (with single pore layer) characterized by SEM are presented in Figure 2 for BL-NAAs and in the supplementary information for the control NAAs structures. A representative top view SEM image of BL-NAAs confirmed the same pore shape and uniform pore size on the surface for BL-NAA_(25/75)_, BL-NAA_(50/50)_, BL-NAA_(75/25)_, NAA_(1),_ and NAA_(2)_ (supplementary information). Table 2 shows the overall pore dimensions of BL-NAAs and NAAs structures. The pore diameters of the top layer for BL-NAA_(25/75)_, BL-NAA_(50/50)_, BL-NAA_(75/25)_, NAA_(1)_ generated by anodization and pore widening process was confirmed to be about 55 nm and similar for all samples. The pore diameters of NAA_(2)_ prepared only by the anodization process are 45 nm in size and the bottom layer pore sizes were confirmed by cross-sectional imaging. Cross-sectional images of BL-NAA revealed two stack layers of cylindrical and vertical ordered nanostructures, the top layer with larger and the bottom layer with smaller pore dimeters was confirmed by a series of SEM images (Figure 2f). The series of cross section images with different magnifications showed three different types of bi-layered structures with thicknesses of 30 ± 1.5 to 32 ± 1.6 μm and different length of top and bottom layers. The length of the top nanoporous layer was 9 ± 0.45 μm for BL-NAA_(25/75)_, 15 ± 0.75 μm for BL-NAA_(50/50)_ and 22 ± 1.1 μm for BL-NAA_(75/25)_. Consequently, the length of the bottom layer was determined as 22 ± 1.1 μm for BL-NAA_(25/75)_, 15 ± 0.75 μm for BL-NAA_(50/50),_ and BL-NAA_(75/25)_, 10 ± 0.5 μm. Table 2 summarized the nanopore dimensions for BL-NAAs. It is observed that the bottom layers have smaller pore diameters which caused funnel like bi-layered structures. The bottom layers of nanostructures, BL-NAA_(25/75)_, BL-NAA_(50/50)_, BL-NAA_(75/25)_ all showed a 45 nm pore diameter. The results of the SEM characterization can be explained due to the fact that the top layer formation of the bilayered structures occurred during the second step of anodization and the second step of anodization is responsible for bottom layer fabrication. It is also understood that the pore-widening process is the main reason that differences arise in pore diameters between the top layer and bottom layer which forms a funnel like structure. This was clearly proved where the control NAA_(1)_ and control NAA_(2)_ platforms revealed a single layer of nanostructure when one step of anodization and pore-widening process was eliminated. 

### 3.2. Characterization and Optimization of RIfS Signals from BL-NAAs and NAAs Platforms

**Figure 3:**
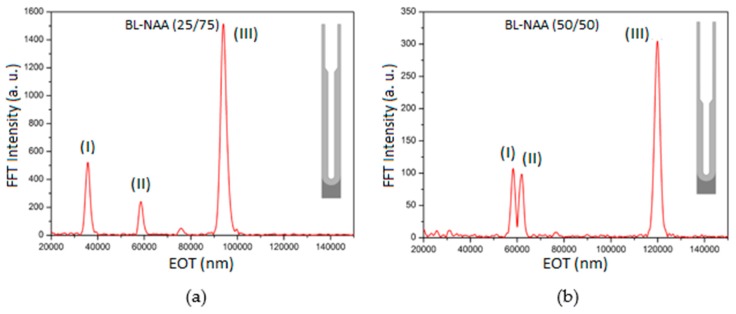

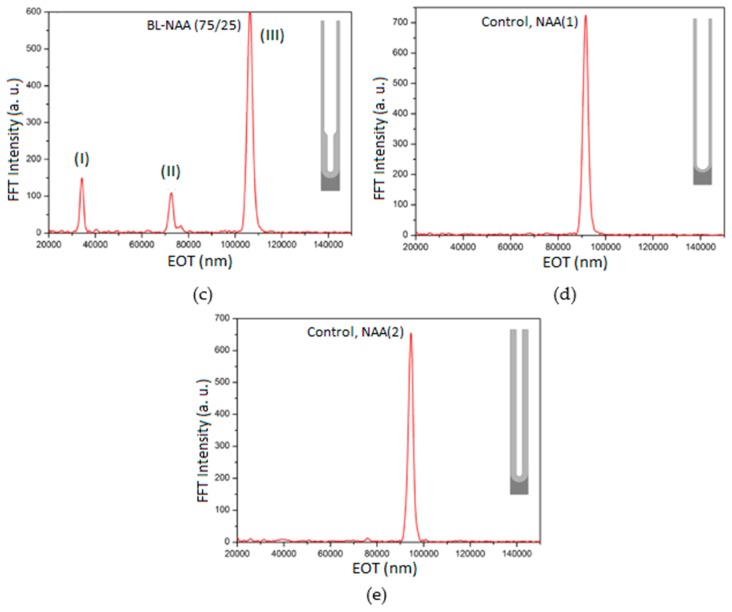
Comparison of FFT spectra of BL-NAAFs and NAAs with single layer and bilayer of nanoporous alumina structure. (**a**) Bi-layer structured of BL-NAA_(25/75)_ produced during (Step1 = 20 h, step 2 = 3 h, 15 min pore widening, and step 3 = 6 h with no further pore widening), resulted in three optical peaks including (I), (II), and (II) various light reflection properties (e.g., FFT intensity and effective optical thickness); (**b**) Bi-layer structured of BL-NAA_(50/50)_ produced during (Step 1 = 20 h, step 2 = 4.5 h, 15 min pore widening, and step 3 = 4.5 h with no further pore widening), resulted in three optical peaks including (I), (II), and (II) various light reflection properties (e.g., FFT intensity and effective optical thickness); (**c**) Bi-layer structured of BL-NAA_(75/25)_ produced during (Step 1 = 20 h, step 2 = 6 h, 15 min pore widening, and step 3 = 3 h with no further pore widening), resulted in three optical peaks including (I), (II), and (II) various light reflection properties (e.g., FFT intensity and effective optical thickness). (**d**,**e**) Single-layer of control NAA_(1)_, and control NAA_(2)_ with a unique FFT.

As confirmed by SEM images, BL-NAA platforms feature a bilayered structure with larger pore diameters from the top, 55 nm, to smaller pore diameter at the bottom, 45 nm, with three different thicknesses of top and bottom layers. The fast Fourier transform (FFT) spectra of BL-NAAs, generated from optical interference pattern (see details of optical interference pattern in Supplementary Information), demonstrated more complex patterns than NAAs with a single layer structure. Figure 3 summarizes the FFT spectra of five different NAA structures analyzed in this study, including BL-NAA_(25/75)_, BL-NAA_(50/50)_, BL-NAA_(75/25)_, NAA_(1),_ and NAA_(2)_. FFT spectra from BL-NAA_(25/75)_, BL-NAA_(50/50)_, BL-NAA_(75/25)_ (Figure 3a–c) feature three characteristic peaks with specific EOT with diverse FFT intensities that are used for sensing in this work. As can be seen, three peaks for BL-NAA_(25/75)_ are observed at EOT_(I)_ = 38,000 ± 1900 nm, EOT_(II)_ = 59,000 ± 2950 nm, and EOT_(III)_ = 95,000 ± 4750 nm, BL-NAA_(50/50)_ showed EOT_(I)_ = 59,000 ± 2950 nm, EOT_(II)_ = 61,000 ± 3050 nm, and EOT_(III)_ = 120,000 ± 6000 nm, BL-NAA_(75/25)_ also showed EOT_(I)_ = 38,000 ± 1900 nm, EOT_(II)_ = 71,000 ± 3550 nm, and EOT_(III)_ = 107,000 ± 5350 nm. FFT peaks for EOT_(I)_, EOT_(II)_, and EOT_(III)_ correspond to the top, bottom, and total layers within the structure of BL-NAA platforms with three levels of light reflection. This result is in agreement with previous studies showing similar optical responses [32]. This phenomenon can be readily used to achieve unique sensing capabilities associated with EOT changes of each of these three peaks. In contrast to BL-NAAs, NAA_(1)_ and NAA_(2)_ showed a single peak in their FFT spectra, which is associated with the EOT of the nanoporous film (Figure 3d,e). Detection of quercetin using HSA-modified BL-NAAs and NAAs through RIfS was used to demonstrate the sensitivity of these optical platforms across all three optical layers. In this process, the EOT change obtained by applying FFT was used as sensing parameter to monitor in real-time the binding events occurring in each of the sensing layers. 

### 3.3. The Evaluation of Bl-NAA and NAA Sensing Platforms for Biomolecules Sensing

**Figure 4:**
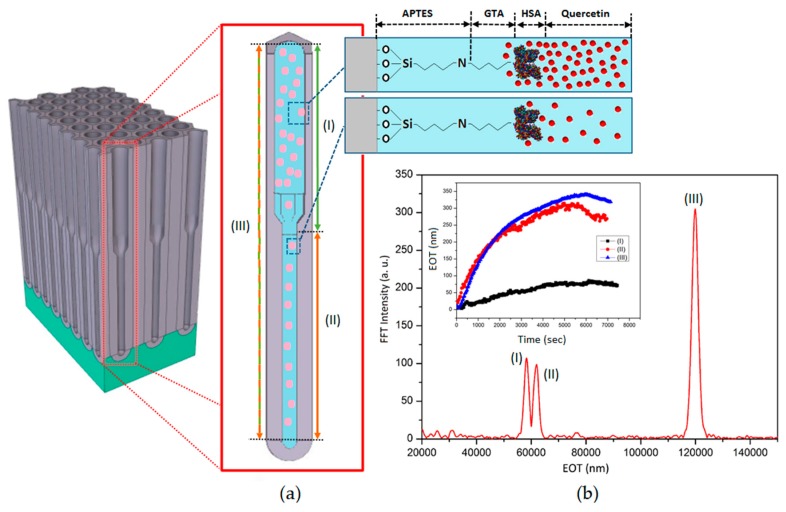
The proposed RIfS sensing concept using BL-NAAs platforms showing changes of EOT signals during surface modification and the preparation of sensing layer (HSA) and sensing analyte molecules (quercetin) showing the binding activity between HSA-modified pore surface and quercetin. (**a**) Schematic illustration of binding event between HSA and quercetin in the environment of fresh PBS (pH = 7.5) in the top and bottom pore layer. BL-NAAs and NAAs were modified with silane molecules before subjecting them to real-time monitoring process; (**b**) typical FFT spectra of BL-NAA_(50/50)_ showing 3 distinguished peaks that are related to two pore layers and used for sensing showing time response RIfS signal that present EOT measurement quercetin (0.375 mM) binding activity in all 3 optical layers simultaneously.

Figure 4 illustrates the proposed molecular distribution and binding reaction of sensing molecules inside larger and smaller pores of BL-NAA and continuous RIfS signals (ΔEOT as the sensing parameter) obtained during different stages of our study to assess the sensing performance of BL-NAA platforms. This sensing process is based on real-time monitoring of EOT of each corresponding nanoporous layers that were monitored through all steps from the fabrication of sensing surface to final sensing of analyte molecules (Figure 4b). The following steps are observed in the figure including (i) amine (-NH_2_) group activation by GTA; (ii) HSA functionalization of inner surface of BL-NAAs and NAAs; and (iii) quercetin binding reaction to HSA. Changes of EOT were observed during GTA, HSA and quercetin flow into the system which means the binding reaction between the molecules and the inner surface of BL-NAAs. Finally, sensing of quercetin molecules revealed stable and higher EOT where all HSA molecules are saturated and no further binding reaction occurred showing the sensing capabilities of this system.

**Figure 5:**
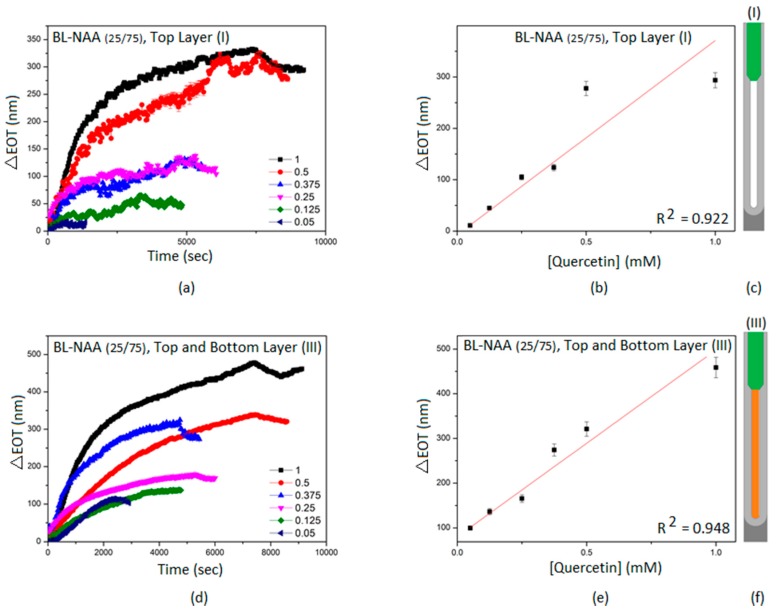
Time response curves showing effective optical thickness (EOT) changes and Linear fitting line for BL-NAA_(25/75)_ (funnel structure with shorter top layer with large pore diameters and longer bottom layer with smaller pore diameters) sensing platforms measured by RIfS as a function of the quercetin concentration (0.05, 0.125, 0.25, 0.375, 0.5, and 1 mM). (**a**) ΔEOT_(I)_ generated from the top structural layer of the BL-NAA_(25/75)_ platform; (**b**) Linear fitting line for BL-NAA_(25/75)_ between ΔEOT_(I)_ and the different concentrations of quercetin (0.05, 0.125, 0.25, 0.375, 0.5, and 1 mM); (**c**) Scheme of the representative optical layer as a function of ΔEOT_(I)_ assessment; (**d**) ΔEOT_(III)_ generated from the top and bottom nanoporous structure of the BL-NAA_(25/75)_ platform; (**e**) Linear fitting line for BL-NAA_(25/75)_ between ΔEOT_(III)_ and the different concentration of quercetin (0.05, 0.125, 0.25, 0.375, 0.5, and 1 mM). (**f**) Scheme of representative optical layer as a function of ΔEOT_(III)_ assessment.

**Figure 6:**
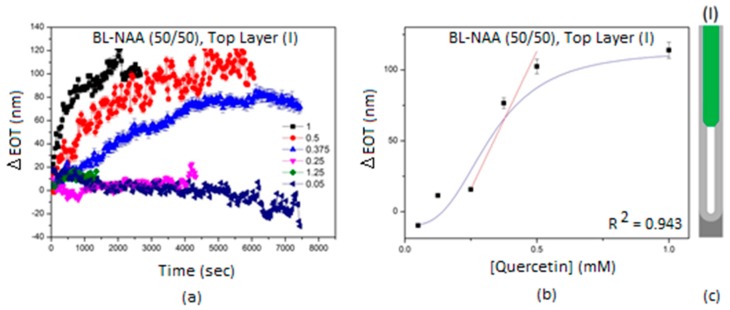

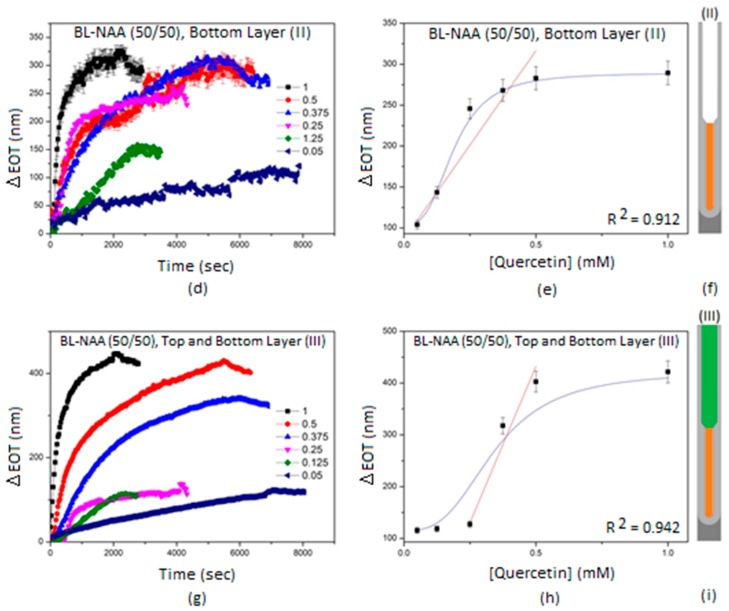
Time response curve showing effective optical thickness changes and sigmoidal curve fitting for BL-NAA_(50/50)_ (funnel structure with equal top layer with large diameters and bottom layer with smaller pore diameters) sensing platforms measured by RIfS as a function of quercetin concentration (0.05, 0.125, 0.25, 0.375, 0.5, and 1 mM). (**a**) ΔEOT_(I)_ generated from the top structural layer of the BL-NAA_(50/50)_ platform; (**b**) Sigmoid fit (blue) and the linear fit (red) for BL-NAA_(50/50)_ between ΔEOT_(I)_ and the different concentration of quercetin (0.05, 0.125, 0.25, 0.375, 0.5, and 1 mM) are presented; (**c**) Scheme of representative optical layer as a function of ΔEOT_(I)_ assessment; (**d**) ΔEOT_(II)_ generated from the bottom structural layer of the BL-NAA_(50/50)_ platform; (**e**) Sigmoid fit (blue) and the linear fit (red) for BL-NAA _(50/50)_ between ΔEOT _(II)_ and the different concentration of quercetin (0.05, 0.125, 0.25, 0.375, 0.5, and 1 mM) are presented; (**f**) Scheme of representative optical layer as a function of ΔEOT_(II)_ assessment; (**g**) ΔEOT_(III)_ generated from the top and bottom nanoporous structure of BL-NAA_(50/50)_ platform; (**h**) Sigmoid fit (blue) and the linear section (red) for BL-NAA_(50/50)_ between ΔEOT _(III)_ and the different concentration of quercetin (0.05, 0.125, 0.25, 0.375, 0.5, and 1 mM) are presented; (**i**) Scheme of representative optical layer as a function of ΔEOT_(III)_ assessment.

**Figure 7:**
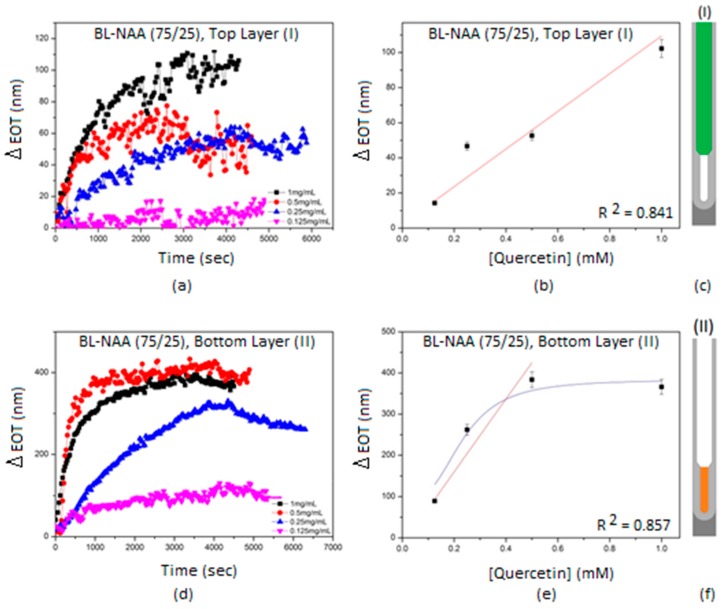

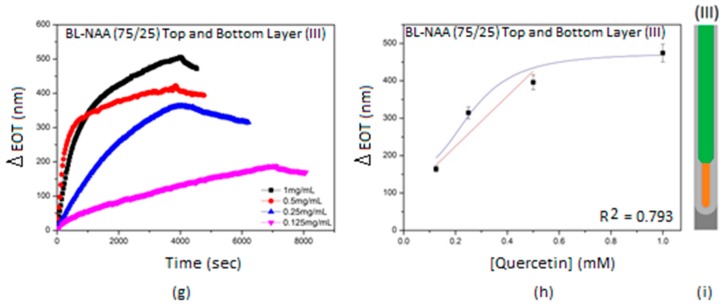
Time response curve showing effective optical thickness changes and fitting curves for BL-NAA_(75/25)_ (funnel structure with longer top layer with large diameters and shorter bottom layer with smaller pore diameters) sensing platforms measured by RIfS as a function of quercetin concentration (0.125, 0.25, 0.5, and 1 mM). (**a**) ΔEOT_(I)_ generated from top structural layer of BL-NAA_(75/25)_ platform; (**b**) Linear fitting line for BL-NAA_(75/25)_ between ΔEOT_(I)_ and the different concentration of quercetin (0.125, 0.25, 0.5, and 1 mM); (**c**) Scheme of representative optical layer as a function of ΔEOT_(I)_ assessment; (**d**) ΔEOT_(II)_ generated from bottom structural layer of BL-NAA_(75/25)_ platform; (**e**) Sigmoid fit (blue) and the linear fit (red) for BL-NAA_(75/25)_ between ΔEOT _(II)_ and the different concentration of quercetin (0.125, 0.25, 0.5, and 1 mM) are presented; (**f**) Scheme of representative optical layer as a function of ΔEOT_(II)_ assessment; (**g**) ΔEOT_(III)_ generated from the top and bottom nanoporous structure of the BL-NAA_(75/25)_ platform; (**h**) Sigmoid fit (blue) and the linear fit (red) for BL-NAA_(75/25)_ between ΔEOT _(III)_ and the different concentration of quercetin (0.05, 0.125, 0.25, 0.375, 0.5, and 1 mM) are presented; (**i**) Scheme of representative optical layer as a function of ΔEOT_(III)_ assessment.

**Figure 8:**
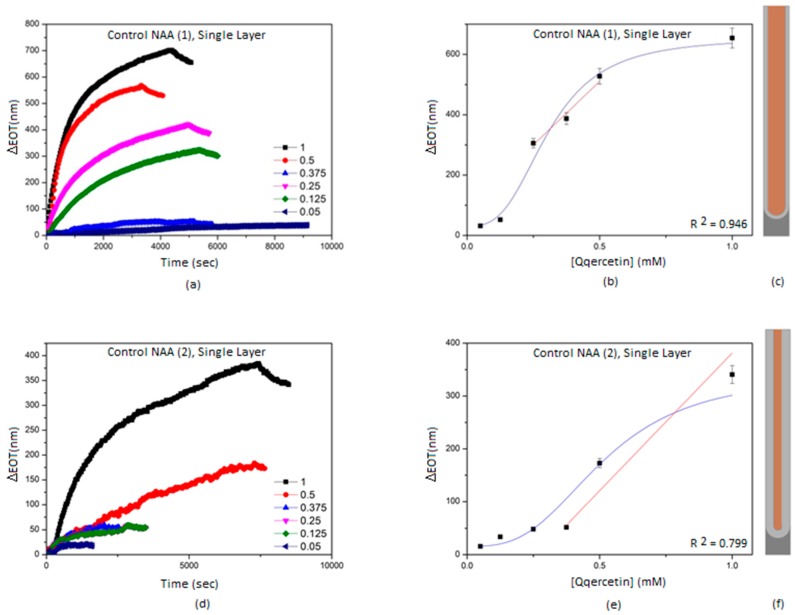
Time response curves showing effective optical thickness changes and fitting curves for control NAA_(1)_ and NAA_(1)_ sensing platforms measured by RIfS as a function of quercetin concentration (0.05, 0.125, 0.25, 0.375, 0.5, and 1 mM). (**a**) ΔEOT generated from single layer nanoporous structure of NAA_(1)_ platform; (**b**) Sigmoid fit (blue) and the linear fit (red) for NAA_(1)_ between ΔEOT and the different concentration of quercetin (0.05, 0.125, 0.25, 0.375, 0.5, and 1 mM) are presented; (**c**) Scheme of representative optical layer as a function of ΔEOT assessment; (**d**) ΔEOT generated from single layer nanoporous structure of NAA_(2)_ platform; (**e**) Sigmoid fit (blue) and the linear fit (red) for NAA_(2)_ between ΔEOT and the different concentration of quercetin (0.05, 0.125, 0.25, 0.375, 0.5, and 1 mM) are presented; (**f**) Scheme of representative optical layer as a function of ΔEOT assessment.

The obtained results for ΔEOT for each analytical solution of quercetin (0.05, 0.125, 0.25, 0.375, 0.5, and 1 mM) using BL-NAAs with different layer thicknesses and control NAAs are displayed and summarized in Figure 5, Figure 6, Figure 7 and Figure 8 and Table 3. The calculation of linear fitting lines between ΔEOT and the concentration of quercetin was used to estimate the sensitivity, S which is the slope of the fitting lines, the low limit of detection, LLOD which is calculated as 3σ according the Equation (2), and the linearity, *R*^2^ which is the correlation coefficient of the fitting line, for each BL-NAA and NAA (Table 3). 

LLOD = 3σ = 3.3 standard error ÷ slope (2)

Figure 5 presents the ΔEOTs of BL-NAA_(25/75)_ platform with a funnel-like structure consisting of a shorter top layer of large pore diameter (Figure 5a) anda longer bottom layer featuring a smaller pore diameter obtained from the top layer I and combined top and bottom layer (Figure 5d). Generally, the ΔEOT_(III)_ signal from combined top and bottom layer (optical layer 3) showed higher values compared to ΔEOT_(I)_ taken from the first layers. The ΔEOT_(I)_ and ΔEOT_(III)_ were calculated with maximum changes of 300 nm and 450 nm, respectively and were obtained for the highest concentration of quercetin 1 mM (Figure 5a,d). Notice that the ΔEOT_(II)_ measurement from the bottom layer is not included during investigation of this type of BL-NAA platform due to the lack of sufficient optical efficiency of the bottom layer of the BL-NAA_(25/75)_ platform which caused failure in the EOT screening. Calibration graphs also showed that the ΔEOT has a linear dependence with quercetin concentrations (Figure 5b,e). Comparative calibration graphs (Figure 5b vs. 5e) also showed a higher precision of measurement taken from optical layer III evidenced by the correlation factors *R*^2^ BL-NAA_(25/75)_ (I) = 0.922 and BL-NAA_(25/75)_ (III) = 0.948 (Table 3). It is seen that layer (III), has a smaller dispersion around the fitting line compared to layer (I), indicating a stronger linear relationship of layer (III) than layer (I) (Figure 5b,e). The sensing performance evaluation of BL-NAA_(25/75)_ resulted in values of *S*_(I)_ = 378 ± 48 (nm mM^−1^) and *S*_(III)_ = 418 ± 43 (nm mM^−1^) for BL-NAA_(25/75)_ (I) and BL-NAA_(25/75)_ (III), respectively. These results showed both optical layer (I) and optical layer (III) acted as highly sensitive sensing areas with the ability of *LLOD*_(I)_ = 0.034 mM and *LLOD*_(III)_ = 0.074 mM quercetin detection. 

Figure 6 summarizes the response time graphs of ΔEOTs of BL-NAA_(50/50)_, platform (funnel structure equal length of top layer of large diameters and longer layer with smaller pore diameters) obtained from layer (I), layer (II), and layer (III) using a series of standard quercetin concentrations. These results were used to make corresponding calibration curves with schematic illustrations of a singular nanopore structure highlighting layer (I) in green color, layer (II) in orange color and layer (III) in green/orange color used for sensing in each graph. The time response graphs, for each optical layer (The ΔEOT_(I)_, ΔEOT_(II)_ and ΔEOT_(III)_) showed different values for the maximum concentration of quercetin (1 mM) starting from 120 nm, 270 nm, and 420 nm, respectively, (Figure 6a,d,g) indicating a different sensitivity for each layer. The sigmoidal curve fitting shows that the ΔEOT has a sigmoidal dependence with the concentration of quercetin (Figure 6b,e,h). However, there are linear correlations amongst quercetin concentrations (i.e., 0.25, 0.375, and 0.5 mM). The dispersion of those data points represents similar *R*^2^ values of the top layer (I), bottom layer (II) and top and bottom layer (III) which are BL-NAA_(50/50)_ (I) = 0.943, BL-NAA_(50/50)_ (II) = 0.912, and BL-NAA_(50/50)_ (III) = 0.942 (Table 3). The sensing performance of BL-NAA_(50/50)_, evaluated by sensitivity graphs, showed values of *S*_(I)_ = 389 ± 66 (nm mM^−1^), *S*_(II)_ = 462 ± 70 (nm mM^−1^), and *S*_(III)_ = 1207 ± 208 *S* (nm mM^−1^) for BL-NAA_(50/50)_ (I), BL-NAA_(50/50)_ (II) and BL-NAA_(50/50)_ (III), respectively which suggest optical layer (III) acted as a highly sensitive sensing area, and optical layer (I) and (II) did not act as high sensitive sensing areas. Optical layer (I) was the least sensitive when compared to two other optical layers. *LLOD*_(I)_ = 0.148 mM, *LLOD*_(II)_ = 0.1 mM, and *LLOD*_(III)_ = 0.168 mM were evaluated for the limit of quercetin detection. The sensitivity of optical layer III compared with results of the funnel with a shorter top layer significantly decreased from *S*_(III)_ = 1207 ± 208 to S_(III)_ = 418 ± 43 S (nm mM^−1^).

Figure 7 summarizes response time graphs of ΔEOTs based on BL-NAA (75/25), platform (funnel structure with longer thickness of top layer of large diameters and shorter layer with smaller pore diameters) obtained from layer (I), layer (II), and layer (III) using BL-NAA (50/50), and a series of standard quercetin concentrations. The pertinent calibration curves are shown of ΔEOT and quercetin concentrations, with schematic illustrations of the singular nanopore structure highlighting layer (I) in green color, layer (II) in orange color and layer (III) in green/orange color. According to the results, the ΔEOT_(I)_, ΔEOT_(II),_ and ΔEOT_(III)_ obtained from the highest quercetin concentrations showed maximum changes of 100 nm, 370 nm, and 460 nm, respectively (Figure 7a,d,g) which suggests the highest sensitivity for optical layer III. The calibration graph shows that ΔEOT has linear dependence with the all concentration of quercetin of layer (I) (Figure 7b). However, there is a sigmoid correlation with the concentrations of the quercetin of layer (II) and layer (III) (Figure 7e,h). The dispersion of data points around the fitting curves for ΔEOT_(I)_ ΔEOT_(II)_ represents a strong correlation between the top layer (I) and bottom layer (II). Evidenced by the calculated *R*^2^ are BL-NAA_(75/25)_ (I) = 0.841, BL-NAA_(75/25)_ (II) = 0.857, and BL-NAA_(75/25)_ (III) = 0.793, presented in Table 3.

The evaluated sensing performance of BL-NAA_(75/25)_ gave values *S*_(I)_ = 107 ± 26 (nm mM^−1^), *S*_(II)_ = 884 ± 245 (nm mM^−1^) and *S*_(III)_ = 670 ± 227 *S* (nm mM^−1^) for BL-NAA_(75/25)_ (I), BL-NAA_(75/25)_ (II) and BL-NAA_(75/25)_ (III), respectively. These results showed that optical layers (III) and (II) acted as highly sensitive sensing areas, and that optical layer (I) did not act as a high sensitive sensing area. *LLOD*_(I)_ = 0.179 mM, *LLOD*_(II)_ = 0.160 mM and *LLOD*_(III)_ = 0.256 mM are evaluated for the limit of quercetin detection. 

Figure 8 summarizes response time ΔEOTs graphs based on the single layer of NAA_(1)_ and NAA_(2)_, used as control corresponding to calibration graphs and schematic illustrations of the singular nanopore structure highlighted in brown color. According to the results, ΔEOT of NAA_(1)_ shows higher values compared to ΔEOT of NAA_(2)_ (Figure 8a,d). These graphs also show that ΔEOTs have a sigmoid dependence with the concentration of quercetin (Figure 8b,e). The dispersion of data points around the curve for the ΔEOT of NAA_(1)_ represents a stronger correlation compared with the ΔEOT for NAA_(2)_, evidenced by R^2^ 0.946 for NAA_(1)_ and 0.799 for NAA_(2)_ (Table 3). The sensing performance evaluation of NAA_(1)_ resulted in a value of *S* = 842 ± 139 (nm mM^−1^). These results showed that the single optical layer of NAA_(1)_ acted as a highly sensitive sensing platform. *LLOD* = 0.188 mM of NAA_(1)_ is evaluated for the limit of quercetin detection. The sensing performance evaluation of NAA_(2)_ resulted in a value of *S* = 521 ± 174 (nm mM^−1^). These results showed the single optical layer of NAA_(2)_ did not act as a highly sensitive sensing platform. The *LLOD* = 0.449 mM of NAA_(2)_ was evaluated for the limit of quercetin detection. Control NAA_(1)_ showed a higher level of sensitivity which means a larger pore diameter can affect the sensing performance. 

**Figure 9:**
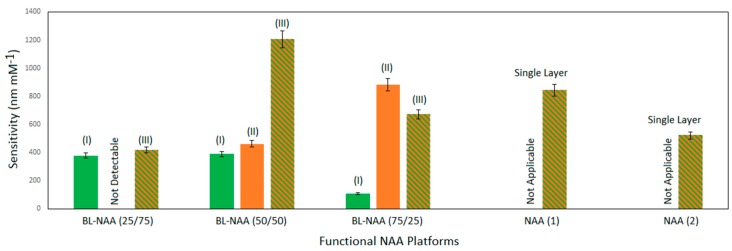
Bar chart summarizing the sensitivities (i.e., the slope of linear fittings shown in Figure 5, Figure 6, Figure 7 and Figure 8) for BL-NAAs and NAAs sensing platforms modified with HSA-quercetin.

The obtained sensitivity results of the overall optical sensing performance for BL-NAAs and NAAs in this study are summarized in Figure 9 and Table 3. According to the data, optical layer (I) of BL-NAA_(25/75)_ showed almost similar *S* with the optical layer (I) of BL-NAA_(50/50)_ whilst optical layer (I) of BL-NAA_(75/25)_ showed a lower *S* than optical layer (I) of BL-NAA_(50/50)_ and BL-NAA_(25/75)_. These results confirmed that the shorter top layer of the bilayered structure has the better sensitivity and stronger optical capability. The optical layer (II) of BL-NAA_(75/25)_ revealed the highest sensitivity compared to optical layers (II) of BL-NAA_(25/75)_ and BL-NAA_(50/50)_ which further leads us to the conclusion that the shorter structural layer in a bilayered structure caused higher sensitivity. This is an important conclusion that should be considered when designing optimal sensing of multilayered NAA structures. Therefore, thick and in-depth fabrication of bilayered nanostructures reduced the optical characteristics of nanostructures, as seen by failed optical screening for optical layer (II) of BL-NAA_(25/75)_. Interestingly, optical layer (III) of BL-NAA_(50/50)_ showed highest sensitivity compared to optical layers (III) of BL-NAA_(25/75)_, BL-NAA_(75/25)_. The value of sensitivities for optical layer (III) of BL-NAA_(50/50)_ was higher compared to the single layer structures used in this study (NAA_(1)_ and NAA_(2)_). Therefore, BL-NAA_(50/50)_ can be the proper choice of optimized bilayered structures due to its capability in multi-point sensing with high sensitivity at each layer. Additionally, control NAA_(1)_ depicted a single path of ΔEOT screening. Control NAAs demonstrated the significant role of pore widening in sensing performance, where a lack of pore widening process diminishes *S*, significantly. Considerably, BL-NAAs could establish the multiple sensing pathways of a diverse range of sensing features. The sensitivities of these five nanoporous anodic alumina platforms are summarized in Figure 9.

## 4. Conclusions

In this work fabrication of bilayered NAA with a funnel-like nanopore structure (larger pore diameter on top and smaller pore diameter on the bottom) was successfully demonstrated using a combination of sequential anodization steps and pore widening process and was confirmed by systematic structural SEM characterizations. The bilayered nanostructures present complex RIfS and FFT spectra, which can be readily and independently used as a sensing pattern. A set of three BL-NAAs platforms with different lengths of porous layers were evaluated to establish the most sensitive pore structures for a quercetin molecule, a model plant flavonol, and detection as a function of three optical layers (i.e., optical layer I, optical layer II, optical layer III). The design of sensing strategy made it possible to establish the effect of each optical layer on the sensitivity of the BL-NAAs using a sensing parameter, that is, changes in the effective optical thickness of the film (ΔEOT). Our analysis revealed that BL-NAA_(50/50)_ has the most sensitive sensing pattern amongst BL-NAAs platforms. However, in comparison between a top and bottom structure, the bottom layer with less thickness features higher sensitivity. These results indicate that all these three layers can be used independently for sensing which means that with different surface chemistries this platform can be used for multi analyte biosensing. We believe these types of bilayered NAA structures, with further development of selective chemistry inside the pores, are a promising platform for the footprint development of multi-point sensing RIfS devices for analysis of complex analyte systems including biological and environmental samples.

## Figures and Tables

**Table 1:** Fabrication protocol of BL-NAAs and NAAs that combines anodization and pore widening steps (BL-NAA_(25/75)_ represent ratio in lengths of pore layers with 25% top layer with larger pore diameters and 75% of layer with layer with smaller pore diameters, BL-NAA_(50/50)_ with equal length of both layers and the BL-NAA_(75/25)_ structure with 75% top layer with larger pore diameters and 25% of layer with layer with smaller pore diameters, and NAA_(1)_ with uniform structures with large pore diameters and NAA_(2)_ with smaller pore diameters.)

Anodized Anodic Aluminum Platforms		Step (1) Anodization (h)	Step (2) Anodization (h)	Pore Widening (min)	Step (3) Anodization (h)
NAAF	BL-NAA_(25/75)_	20	3	15	6
	BL-NAA_(50/50)_	20	4.5	15	4.5
	BL-NAA_(75/25)_	20	6	15	3
NAA	NAA_(1)_	20	9	15	N/A ^1^
	NAA_(2)_	20	9	N/A^1^	N/A ^1^

^1^ N/A: Not applied.

**Table 2:** Pore dimensions of BL-NAAs and NAAs structures.

Anodized Anodic Aluminum Platforms		Top Layer Thickness (μm)	Bottom Layer Thickness (μm)	Nanopore thickness (μm)	Pore Diameter (Top/Bottom Layer) (nm)
NAAF	BL-NAA_(25/75)_	9 ± 0.45	22 ± 1.1	30 ± 1.5	55 ± 2.75/45 ± 2.25
	BL-NAA_(50/50)_	15 ± 0.75	15 ± 0.75	30 ± 1.5	55 ± 2.75/45 ± 2.25
	BL-NAA_(75/25)_	22 ± 1.1	10 ± 0.5	32 ± 1.6	55 ± 2.75/45 ± 2.25
NAA	NAA_(1)_	N/A^1^	N/A^1^	22 ± 1.1	55 ± 2.75
	NAA_(2)_	N/A^1^	N/A^1^	23.5 ± 1.17	45 ± 2.25

^1^ N/A: Not applicable.

**Table 3:** Result of sensing performance of BL-NAAs and NAAs by ∆EOT measurement.

Sample	Optical Layer	*S* (nm mM^−1^)	*LLOD* (mM)	*R*^2^
BL-NAA_(25/75)_	(I)	378 ± 48	0.034	0.922
	(II)	ND^1^	ND^1^	ND^1^
	(III)	418 ± 43	0.074	0.948
BL-NAA_(50/50)_	(I)	389 ± 66	0.148	0.943
	(II)	462 ± 70	0.1	0.912
	(III)	1207 ± 208	0.168	0.942
BL-NAA_(75/25)_	(I)	107 ± 26	0.179	0.841
	(II)	884 ± 245	0.160	0.857
	(III)	670 ± 227	0.256	0.793
NAA_(1)_	Single layer	842 ± 139	0.188	0.946
NAA_(2)_	Single layer	521 ± 174	0.449	0.799

^1^ ND means Not Detectable.

## Supplementary Materials

The following are available online at http://www.mdpi.com/1424-8220/18/2/470/s1, Fabrication and optimization of nanoporous anodic alumina structures as multi-point interferometric sensing platform.
