# Supplementary material for: Fabrication and Optimization of Bilayered Nanoporous Anodic Alumina Structures as Multi-Point Interferometric Sensing Platform
Source: Sensors (Basel). 2018 Feb 6;18(2):470. doi: 10.3390/s18020470 (PMC5855889; doi:10.3390/s18020470)
Supplement: Supplementary file 1 [file sensors-18-00470-s001.pdf]

Supplementary Materials

Fabrication and Optimization of Bilayered Nanoporous Anodic Alumina Structures as Multi-Point Interferometric Sensing Platform

Mahdieh Nemati [1], Abel Santos [1,2,3] and Dusan Losic [1,*]

[1] School of Chemical Engineering, The University of Adelaide, Engineering North Building, 5005 Adelaide, Australia ;
[2] Institute for Photonics and Advanced Sensing (IPAS), The University of Adelaide, 5005 Adelaide, Australia;
[3] ARC Centre of Excellence for Nanoscale BioPhotonics (CNBP), The University of Adelaide, 5005 Adelaide, Australia;
*****Correspondence: dusan.losic@adelaide.edu.au

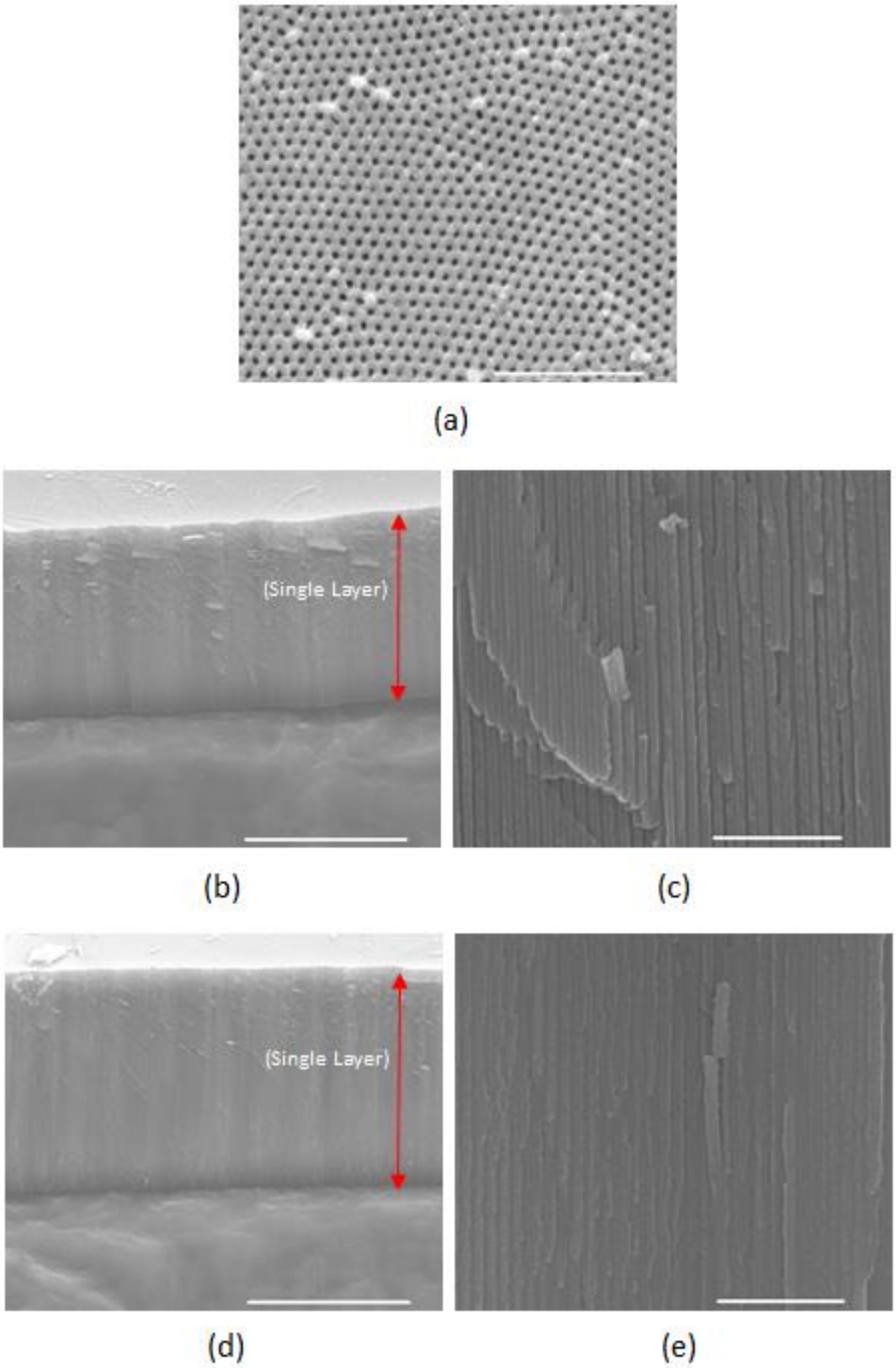

Figure S1. (a) Top view SEM image of a BL-NAA/NAA (scale bar= 1μm) showing typical highly ordered NAA pore structures. (h) Cross-section view of NAA $_{(1)}$, scale bar is 20 μm. (i) Cross-section view of NAA $_{(1)}$, scale bar is 1 μm. (j) Cross-section view of NAA $_{(2)}$, scale bar is 20 μm. (k) Cross-section view of NAA $_{(2)}$, scale bar is 1 μm. Magnified view of red squares in (c), (e), and (g) show the connection of structural layers where pore diameter of each layer is different. Top layer (1), bottom layer (II) and single layers are marked on images.

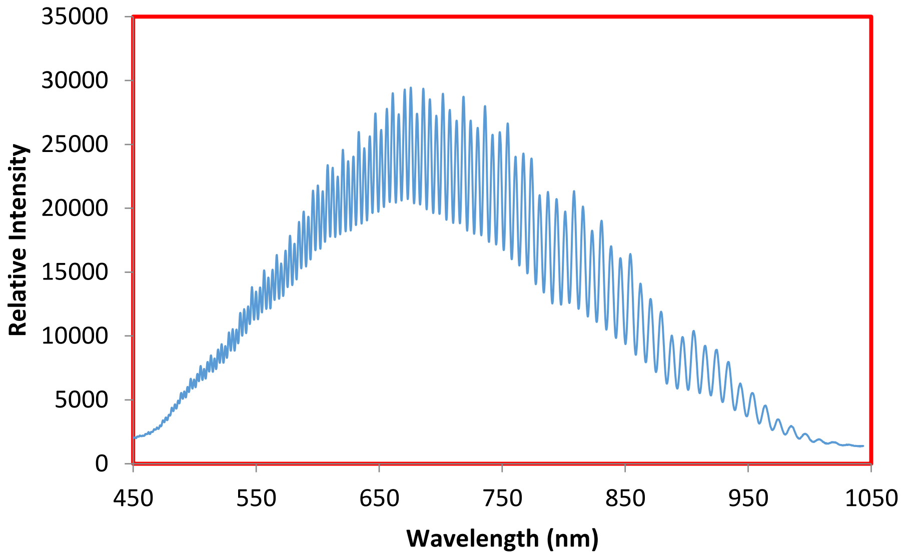
35000
30000
25000
20000
15000
10000
5000
0
Relative Intensity
450
550
650
750
850
950
1050
Wavelength (nm)

(a)

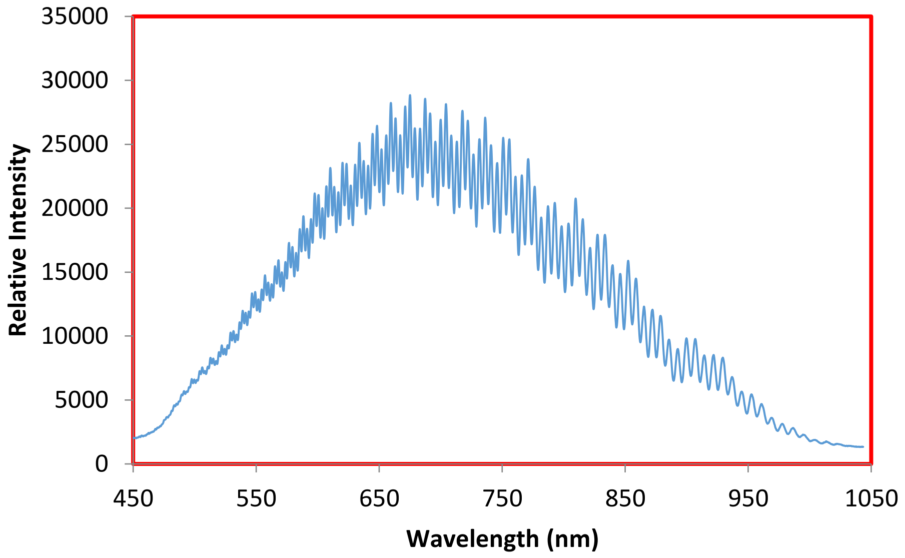
35000
30000
25000
20000
15000
10000
5000
0
Relative Intensity
450
550
650
750
850
950
1050
Wavelength (nm)

(b)

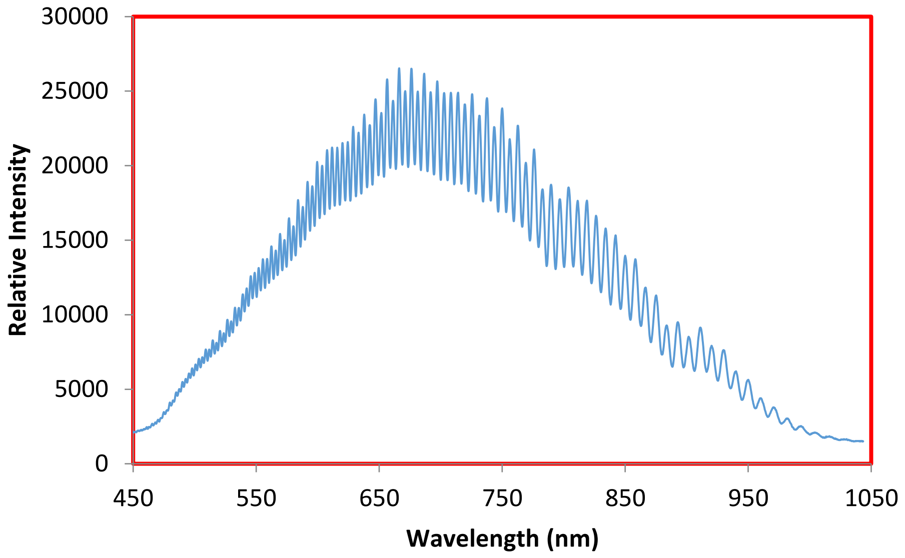
30000
25000
20000
15000
10000
5000
0
Relative Intensity
450
550
650
750
850
950
1050
Wavelength (nm)

(c)

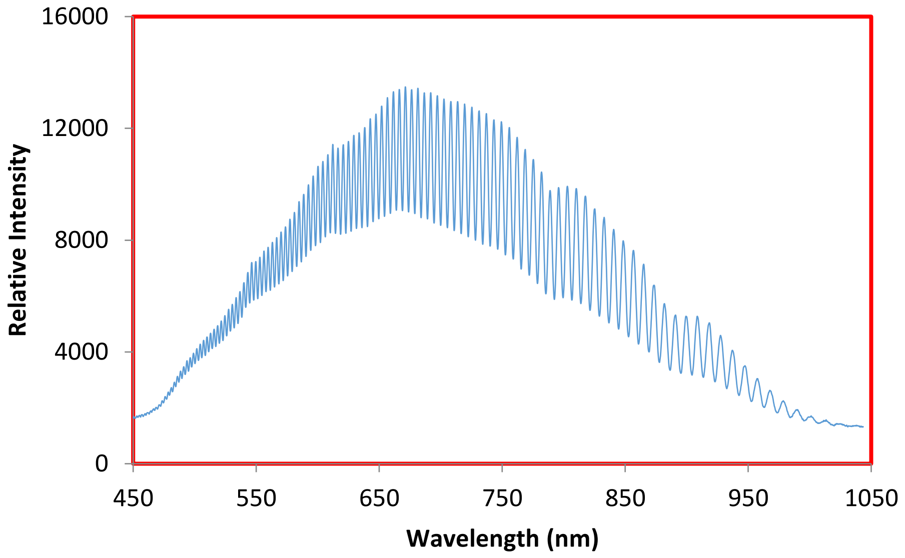
16000
12000
8000
4000
0
Relative Intensity
450
550
650
750
850
950
1050
Wavelength (nm)

(d)

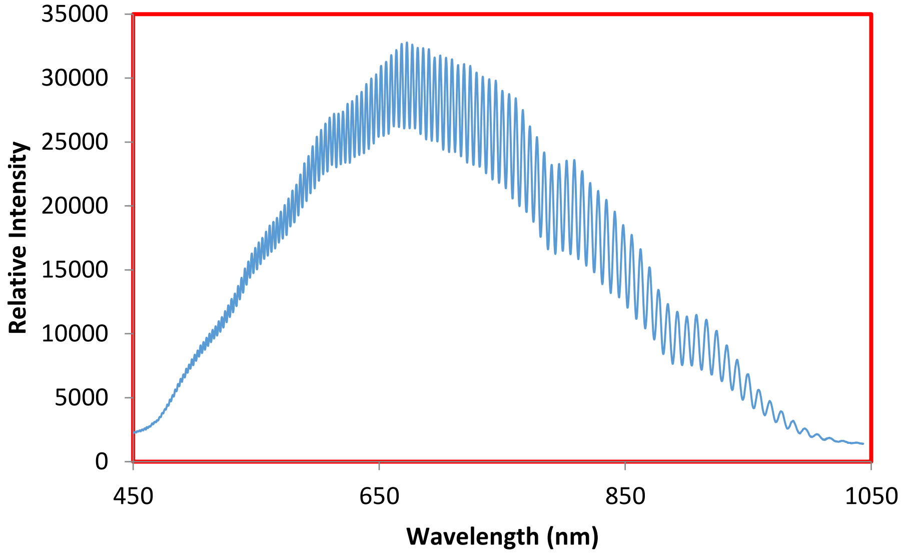

(e)

Figure S2. Optical interference pattern generated from Fabry-Pérot effect for all types of BL-NAAs and NAAs in this study. (a) BL-NAA$_{(25/75)}$, (b) BL-NAA$_{(75/25)}$, (c) BL-NAA$_{(50/50)}$, (d) NAA$_{(1)}$, (e) NAA$_{(2)}$. All optical films presented uniform oscillation series in their RIfS spectrum.
